# Serum Lipid Concentrations, Prevalence of Dyslipidemia, and Percentage Eligible for Pharmacological Treatment of Korean Children and Adolescents; Data from the Korea National Health and Nutrition Examination Survey IV (2007–2009)

**DOI:** 10.1371/journal.pone.0049253

**Published:** 2012-12-14

**Authors:** Seung Yang, Jin Soon Hwang, Hong Kyu Park, Hae Sang Lee, Hae Soon Kim, Eun Young Kim, Jung Sub Lim

**Affiliations:** 1 Department of Pediatrics, Hallym University College of Medicine, Seoul, Korea; 2 Department of Pediatrics, Ajou University School of Medicine, Ajou University Hospital, Suwon, Korea; 3 Department of Pediatrics, Ewha Woman's University School of Medicine, Seoul, Korea; 4 Department of Pediatrics, College of Medicine Chosun University, Gwangju, Korea; 5 Department of Pediatrics, Korea Cancer Center Hospital, Seoul, Korea; FuWai hospital, Chinese Academy of Medical Sciences, China

## Abstract

**Objectives:**

Dyslipidemia is one of the important modifiable risk factors for cardiovascular disease. Thus, to know the prevalence of dyslipidemia is the 1^st^ step to make guidelines of screening and management plan. Although, American Academy of Pediatrics updated the guidelines for lipid in childhood, Asian study is rare.

**Methods:**

The authors aimed to make a reference of each serum lipid level of Korean children and adolescents (2,363 subjects aged 10 to 18 years) from the data of Korea National Health and Nutrition Examination Survey IV (2007–2009).

**Results:**

The mean serum concentrations for total cholesterol (TC), low-density lipoprotein cholesterol (LDL-C), triglycerides (TG), and high-density lipoprotein cholesterol (HDL-C) were 158 mg/dL, 90 mg/dL, 90 mg/dL, and 49 mg/dL, respectively. The 95th percentile values for TC, LDL-C, and TG were 203 mg/dL, 129 mg/dL, and 185 mg/dL, respectively. The 5th percentile value for HDL-C was 36 mg/dL. The prevalence of hypercholesterolemia, high LDL-C, high TG, and low HDL-C was 6.5%, 4.7%, 10.1%, and 7.1%, respectively. Considering the risk factors such as obesity, hypertension, smoking, and diabetes, approximately 0.41% of the subjects were potentially eligible for pharmacological treatment.

**Conclusions:**

This information may be useful in not only Korean but also Asian planning programs for the prevention of cardiovascular disease through lipid control from childhood.

## Introduction

Cardiovascular disease (CVD) is a major cause of morbidity and mortality worldwide, including Korea [1], [2]. Although CVD does not usually develop until the fourth decade of life, it is well known that atherosclerosis develops from childhood [3]–[5]. The initial stages of atherosclerosis and its progression are associated with dyslipidemia [6]. Increased total cholesterol (TC), low-density lipoprotein cholesterol (LDL-C), triglyceride (TG), decreased high-density lipoprotein cholesterol (HDL-C) are well-known risk factors associated with cardiovascular disease [7]–[10]. Furthermore, dyslipidemia has been found to be closely related to other cardiovascular risk factors, such as hypertension, obesity, and smoking status, not only in adults but also in children and adolescents [4], [7]. With this context, the American Academy of Pediatrics (AAP) recently released a report on lipid screening in childhood [11].

Historically, the prevalence of CVD in Korea has been much lower than that in Western countries. However, recently, the CVD-associated mortality rate in Korea increased up to 27.6%, comparable to that in the United States [2]. Furthermore, the prevalence of obesity and metabolic syndrome, which are risk factors for CVD, in Korea has steadily increased and is now comparable to that in the United States [10], [12]. This is believed to be due to recent changes promoting a western lifestyle, especially with regard to dietary habits, among the Korean people [12], [13]. Thus, lipid-profile screening and detection of dyslipidemia in Korean children and adolescents is important for preventing CVD in their later life. However, nationwide studies of pediatric dyslipidemia are scarce, especially in Asian countries. Data on serum lipid concentrations of Asian children and adolescents are even absent in the prevalence data from the United States [14], [15].

The objectives of this study were as follows: (1) to determine the serum lipid concentrations of Korean children and adolescents aged 10 to 18 years; (2) to estimate the national prevalence of pediatric dyslipidemia; and (3) to estimate the number of children and adolescents eligible for pharmacological treatment using the approach presented in the AAP report.

## Methods

### Study population and data collection

This study was performed using data acquired in the Fourth Korea National Health and Nutrition Examination Survey (KNHANES IV, 2007–2009). These surveys have been conducted periodically since 1998 to assess the health and nutritional status of the non-institutionalized civilian population of Korea. KNHANES IV was a cross-sectional and nationally representative survey with a multistage and stratified sampling design conducted by the Division of Chronic Disease Surveillance, Korea Centers for Disease Control and Prevention. Total 31,705 individuals (11,520 families from 500 sectors based on region and housing) were included in KNHANES IV; among them, 74.5% families participated in health surveillance and blood sampling. A total of 2753 subjects aged 10 to 18 years (representing 5,502,245 individuals) were identified as potential subjects for this study. All subjects and their parents were interviewed at home after informed consent and undergo various examinations including blood sampling. Those with incomplete data for a standardized physical examination, blood pressure (BP) assessments, and laboratory tests, including whole lipid profile, and anthropometric measures and those who were currently taking blood cholesterol-lowering medications were excluded. Thus, the final analytical sample consisted of 2,363 subjects (1,245 boys, 1,118 girls) who were at least 8 h of fasting state.

The blood samples were taken by skilled nurse in mobile vehicle and transported daily to the Central Laboratory (NEODIN Medical Institute, Seoul, Korea). In 2007, serum TC, HDL-C, and TG concentrations were measured enzymatically by using ADIVIA1650 (Siemens/USA) with commercial reagents (CHOL, Siemens/USA; TRIG, Siemens/USA; HDL, Siemens/USA). In 2008–2009, TC, HDL-C, and TG concentrations were enzymatically measured using Hitachi Automatic Analyzer 7600 (Hitachi/Japan) with reagents (Pureauto SCHO-N, DAIICHI/Japan; CHOLESTEST N HDL, DAIICHI/Japan; Pureauto S TG-N, DAIICHI/Japan) by NEODIN Medical Institute.

The LDL-C were calculated with the Friedewald equation (LDL-C = TC−[HDL-C+(TG÷5)]) [16]. Since the LDL-C is calculated on the basis of the measured concentrations of HDL-C and TG, measurement needs to be adequate. Therefore, commutable frozen serum samples were prepared according to the C37-A guideline of the Clinical and Laboratory Standards Institute (CLSI) and analyzed at both NEODIN Medical Institute and CDC's Lipid Reference Laboratory. For accuracy, samples with TG greater than 400 mg/dL samples are also excluded.

### Definition of each criterion

The prevalence of dyslipidemia was assessed according to the NECP and AHA report [5], [17]. The cut-off of each dyslipidemia was as follows: TC concentration, ≥200 mg/dL(5.172 mmol/L); LDL-C, ≥130 mg/dL(3.3618 mmol/L); TG, >150 mg/dL(1.6935 mmol/L); and HDL-C, <35 mg/dL(0.9051 mmol/L). The 1 mg/dL of TC, LDL-C, and HDL-C corresponds to 0.02586 mmol/L and the 1 mg/dL of TG corresponds to 0.01129 mmol/L. According to the AAP Lipid Screening guidelines, pharmacological management can be considered when of LDL-C exceed 190 mg/dL. In the presence of risk factors such as hypertension, obesity, or smoking, pharmacological management can be considered when LDL-C exceed 160 mg/dL. In diabetes mellitus, pharmacological management can be considered if LDL-C is equal to or exceeds 130 mg/dL. High BP in Korean children and adolescents was defined as age- and sex-adjusted BP greater than the 95th percentile [18]. Obesity was defined as body mass index (BMI) greater than the 95th percentile or BMI greater than 25, determined from the 2000 Korean CDC growth charts [19]. Smoking status was assessed by administering a questionnaire to subjects aged more than 12 years.

### Statistical analysis

All data were analyzed using SPSS 17.0 for Windows (SPSS Inc., Chicago, IL, USA). The prevalence of each type of dyslipidemia was estimated by incorporating weights to avoid bias. Weights were created to account for the complex survey design, non-response, and post-stratification. The data are presented as the mean ± SD (or SE). The authors calculated mean and percentile values for TC, LDL-C, TG, and HDL-C concentrations. To compare the means between the sex and among age groups, Student's t test and ANOVA were used. Differences in the prevalence of dyslipidemia as a function of obesity and hypertension status were assessed using chi-square tests. Odds ratios (OR) were used to estimate the relative risk for abnormal lipid concentrations. P-values of <0.05 were considered significant.

## Results

Among the 2,363 subjects, 450 (19.2%) had hypertension, 341 (14.4%) were obese, 26 (1.1%) were current smokers, and 2 girls (0.09%) were newly identified diabetes. Obese subjects were significantly more likely to have at least one abnormal lipid concentration (OR = 3.465 [95% CI, 3.448, 3.483]) and subjects with hypertension were more likely to have at least one abnormal lipid concentration (OR = 1.474 [95% CI, 1.466, 1.482]).

**Figure 1:**
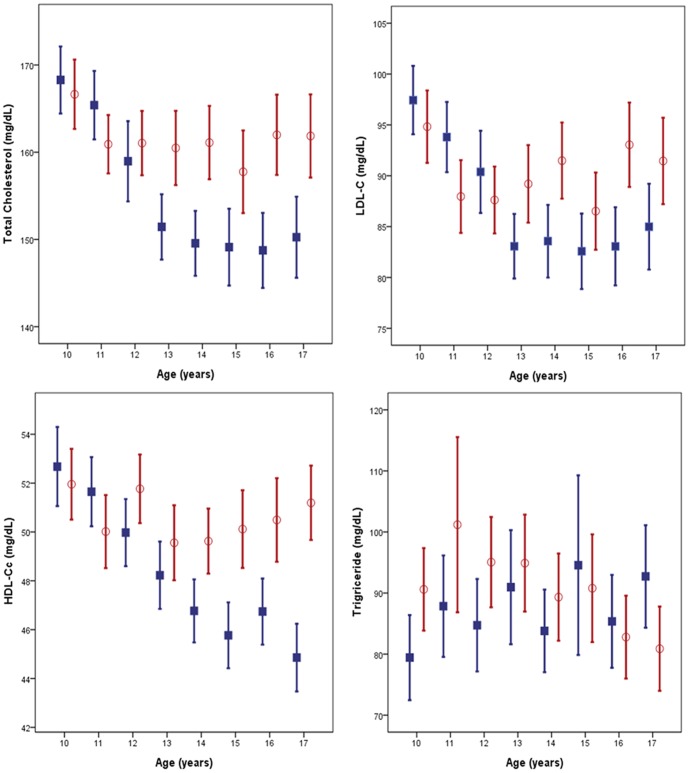
Change of each lipid concentration aged 10 to 18 years (Mean and 95% CI). Blue line and filled square represent boys and Red line and empted circles represent girls. Abbrevation: LDL-C, Low-density lipoprotein cholesterol ; HDL-C, High density lipoprotein cholesterol;.

The age- and sex-stratified mean and percentile values for serum TC are presented in Table 1. The 50th percentile of the TC concentration was 156 mg/dL and the 95th percentile was 203 mg/dL. Overall, girls had a higher mean TC than boys did (161 mg/dL vs. 155 mg/dL, *P*<0.001). Children aged 10–11 years had a mean TC concentration of 166 mg/dL, which was significantly higher than that for 12–18 years (*P*<0.001). Furthermore, there was a significant decrease in mean TC from ages 11–12 years to 13–14 years only in boys (*P*<0.001) (Fig. 1a). Age- and sex-stratified mean and percentile values for LDL-C concentrations are presented in Table 2. Girls had a higher mean LDL-C than boys did (92 mg/dL vs. 89 mg/dL, *P*<0.001), and the mean LDL-C decreased from 12 years of age and then stabilized subsequently only in boys. Girls showed relatively stable LDL-C (Fig. 1b). Age- and sex-stratified mean and percentile values for HDL-C concentrations are presented in Table 3. HDL-C concentrations were relatively constant among age groups for girls aged 10 to 18 years. However, mean HDL-C fell 6 mg/dL from ages 10–12 years to 14–18 years in boys. Thus, girls have higher mean HDL-C than boys did after 14 years of age (*P*<0.001) (Fig. 1c). In addition, the 5th percentile of HDL-C in boys aged 15–18 years was 33 mg/dL, while in girls of the same age group, the corresponding value was 37 mg/dL. The age- and sex-stratified mean and percentile values for TG concentrations are presented in Table 4. The TG concentration showed high variance and no significant difference among age groups, except for the significant difference between the subjects aged 17–18 years and those aged 11–12 years (*P*<0.05). Mean TG values ranged from 81 to 101 mg/dL for boys and 80 to 105 mg/dL for girls according to the age group. The peak TG concentration was noted in the 15–16-year age group in boys and the 11–12-year age group in girls (Fig. 1d).

**Table 1:** Serum Total Cholesterol Concentrations among Korean Children and Adolescents Aged 10–18 years, KNHANES* IV( 2007–2009).

					Percentiles, mg/dL**						
		N	WN	Mean	5	10	25	50	75	90	95
Total cholesterol											
Total		2,363	4,701,322	158	119	126	139	156	173	193	203
Boys		1,245	2,500,720	155	115	123	136	152	171	191	200
	Age, y										
	10–11	159	278,085	168	134	137	149	166	181	202	220
	11–12	173	288,457	165	132	134	145	163	184	200	210
	12–13	179	318,846	159	116	126	145	157	177	194	207
	13–14	175	281,038	152	117	126	135	148	166	185	197
	14–15	167	300,752	150	116	119	131	148	166	180	194
	15–16	139	376,257	149	109	116	129	148	167	179	193
	16–17	136	354,240	148	104	117	135	145	164	180	196
	17–18	117	303,046	150	110	118	129	148	169	186	200
Girls		1,118	2,200,602	161	125	131	143	160	176	194	205
	Age, y										
	10–11	143	256,849	166	129	136	144	166	183	194	207
	11–12	153	272,405	161	129	133	146	160	175	191	198
	12–13	152	263,131	161	127	132	147	160	177	192	201
	13–14	140	251,921	160	125	131	143	160	172	192	201
	14–15	156	262,838	160	121	126	139	160	177	196	200
	15–16	134	336,754	158	121	125	138	156	172	194	207
	16–17	119	282,015	162	126	131	145	160	178	194	203
	17–18	121	274,689	162	122	132	146	156	174	199	209

* KNHANES, Korean National Health and Nutrition Examination Survey.

** 1 mg/dL = 0.02586 mmol/L; 1 mmol/L = 38.66976 mg/dL.

WN; Weighted Number.

**Table 2:** Serum Low-Density Lipoprotein Cholesterol Concentrations among Korean Children and Adolescents Aged 10–18 years, KNHANES* IV( 2007–2009).

					Percentiles, mg/dL**						
		N	WN	Mean	5	10	25	50	75	90	95
Low-density lipoprotein cholesterol											
Total		2,363	4,701,322	90	57	64	75	89	104	120	129
Boys		1,245	2,500,720	89	54	62	73	87	104	119	127
	Age, y										
	10–11	159	278,085	99	62	75	86	99	110	126	135
	11–12	173	288,457	96	66	70	78	97	111	125	131
	12–13	179	318,846	92	56	64	79	89	103	124	132
	13–14	175	281,038	85	57	62	72	81	93	108	125
	14–15	167	300,752	87	53	60	72	84	100	111	124
	15–16	139	376,257	83	51	55	68	82	98	113	117
	16–17	136	354,240	85	49	58	71	84	98	111	125
	17–18	117	303,046	87	53	60	68	85	105	121	126
Girls		1,118	2,200,602	92	60	67	77	90	106	121	132
	Age, y										
	10–11	143	256,849	96	57	69	79	95	112	122	129
	11–12	153	272,405	91	59	68	80	90	103	112	120
	12–13	152	263,131	90	55	66	76	91	101	112	126
	13–14	140	251,921	92	63	66	78	89	101	123	133
	14–15	156	262,838	92	57	64	77	92	106	121	130
	15–16	134	336,754	90	60	66	74	86	104	120	134
	16–17	119	282,015	95	60	65	78	94	109	125	131
	17–18	121	274,689	94	60	70	77	91	107	128	143

* KNHANES, Korean National Health and Nutrition Examination Survey.

** 1 mg/dL = 0.02586 mmol/L; 1 mmol/L = 38.66976 mg/dL.

WN; Weighted Number.

**Table 3:** Serum High- Density Lipoprotein Cholesterol Concentrations among Korean Children and Adolescents Aged 10–18 years, KNHANES* IV( 2007–2009).

					Percentiles, mg/dL**						
		N	WN	Mean	5	10	25	50	75	90	95
High-density lipoprotein cholestetol											
Total		2,363	4,701,322	49	36	38	43	49	55	61	66
Boys		1,245	2,500,720	48	35	37	42	48	54	61	64
	Age, y										
	10–11	159	278,085	52	39	41	45	50	58	66	73
	11–12	173	288,457	52	37	39	46	51	59	62	65
	12–13	179	318,846	51	37	39	44	50	56	63	67
	13–14	175	281,038	49	35	38	42	48	55	62	66
	14–15	167	300,752	47	35	37	39	45	54	59	62
	15–16	139	376,257	46	34	37	41	45	50	56	58
	16–17	136	354,240	46	34	37	41	46	52	56	60
	17–18	117	303,046	46	33	35	40	45	51	55	58
Girls		1,118	2,200,602	50	37	39	44	50	56	63	66
	Age, y										
	10–11	143	256,849	51	37	39	45	51	58	63	66
	11–12	153	272,405	50	37	38	43	49	55	62	68
	12–13	152	263,131	52	38	41	47	52	59	63	66
	13–14	140	251,921	50	37	38	42	50	56	61	66
	14–15	156	262,838	49	37	39	43	48	55	61	64
	15–16	134	336,754	49	33	37	43	48	57	64	66
	16–17	119	282,015	51	38	40	44	49	56	62	67
	17–18	121	274,689	51	37	42	46	51	56	63	66

* KNHANES, Korean National Health and Nutrition Examination Survey.

** 1 mg/dL = 0.02586 mmol/L; 1 mmol/L = 38.66976 mg/dL, WN; Weighted Number.

**Table 4:** Serum Triglycerides Concentrations among Korean Children and Adolescents Aged 10–18 years, KNHANES* IV( 2007–2009).

					Percentiles, mg/dL**						
		N	WN	Mean	5	10	25	50	75	90	95
Triglyceride											
Total		2,363	4,701,322	90	37	42	56	77	106	150	185
Boys		1,245	2,500,720	88	35	41	53	73	103	148	189
	Age, y										
	10–11	159	278,085	82	31	38	48	71	101	136	180
	11–12	173	288,457	86	33	38	51	70	101	152	203
	12–13	179	318,846	86	37	42	53	70	97	141	186
	13–14	175	281,038	91	34	37	50	74	111	161	219
	14–15	167	300,752	81	34	42	54	72	100	134	157
	15–16	139	376,257	101	37	40	54	74	103	146	228
	16–17	136	354,240	84	36	41	56	76	102	152	171
	17–18	117	303,046	91	40	45	62	80	104	160	189
Girls		1,118	2,200,602	92	39	45	59	80	110	151	181
	Age, y										
	10–11	143	256,849	93	42	47	62	81	118	156	183
	11–12	153	272,405	105	38	44	59	93	133	158	186
	12–13	152	263,131	96	42	47	68	85	110	150	199
	13–14	140	251,921	96	45	50	65	86	112	146	220
	14–15	156	262,838	92	39	47	57	77	113	147	198
	15–16	134	336,754	93	43	45	58	80	109	154	174
	16–17	119	282,015	82	37	44	56	73	99	152	169
	17–18	121	274,689	80	37	41	55	72	95	125	165

* KNHANES, Korean National Health and Nutrition Examination Survey.

** 1 mg/dL = 0.01129 mmol/L; 1 mmol/L = 88.57396 mg/dL.

WN; Weighted Number.

Among all subjects, 19.7% had at least one abnormal lipid concentration according to the cutoff points of the NCEP and AHA (Table 5). The estimated prevalence of hypercholesterolemia (>200 mg/dL) was 6.5%. The prevalence of high LDL-C (>130 mg/dL) was 4.7%. The prevalence of high TG (>150 mg/dL) and low HDL-C (<35 mg/dL) was 10.1% and 7.1%, respectively. The prevalence of hypercholesterolemia and high LDL-C was greater in girls than in boys (7.4% vs. 5.8% and 5.5% vs. 4.1%, respectively, *P*<0.001) but lower low HDL-C prevalence (5.5% vs. 8.5%, *P*<0.001).

**Table 5:** Prevalence of Dyslipidemia among Korean Children and Adolescents Aged 10 18 years, according to Guidelines from the NCEP.

		Boys	Girls	Total
Total Cholesterol >200 mg/dL	N	75	85	160
	WN	114,053 (5.8%)	161,925 (7.4%)	305,978 (6.5%)
LDL-C >130 mg/dL	N	58	63	121
	WN	102,111 (4.1%)	120,719 (5.5%)	222,830 (4.7%)
Triglycerides >150 mg/dL	N	120	110	230
	WN	245,455 (9.8%)	227,106 (10.3%)	472,561 (10.1%)
HDL-C <35 mg/dL	N	92	55	147
	WN	212,949 (8.5%)	120,326 (5.5%)	333,275 (7.1%)
LDL-C >190 mg/dL	N	1	1	2
	WN	2,663 (0.11%)	1,293(0.06%)	3,956 (0. 09%)
LDL-C >160 mg/dL with hypertension, obesity, or smoking	N	3	6	9
	WN	5,086 (0.24%)	10,614 (0.48%)	15,700 (0.34%)
Diabetes Mellitus with LDL-C ≥130 mg/dL	N	0	0	0

HDL-C, high density lipoprotein cholesterol; LDL-C, low density lipoprotein cholesterol;

WN, Weighted number.

Among the 2,363 subjects, 2 subjects (1 boys and 1 girl) had an LDL-C >190 mg/dL. Nine subjects (3 boys and 6 girls) with other risk factors (hypertension, obesity, or smoking) had an LDL-C >160 mg/dL. No subject who newly diagnosed diabetes showed elevated LDL-C (≥130 mg/dL). Weighted prevalence of each criterion was 0.09%, 0.34%, and 0.00% respectively. Thus, almost 0.41% of Korean adolescents were eligible for pharmacological treatment.

## Discussion

This is the first Asian study to estimate the percentage of children and adolescents, including those with hypertension and diabetes, eligible for pharmacological treatment according to AAP guidelines. The prevalence of hypercholesterolemia, high LDL-C, high TG, and low HDL-C concentrations among Korean children and adolescent is 6.5%, 4.7%, 10.1%, and 7.1%, respectively. Twenty percent had at least one type of dyslipidemia. Furthermore, almost 0.41% of children and adolescents would qualify for lipid-lowering pharmacological treatment according to the AAP guidelines.

Several major studies such as Lipid Research Clinics Prevalence Study and NHANES have provided age- and sex-specific references for concentrations of lipids in children and adolescents in the United States [14], [15]. These studies also showed that sex, race, and ethnic differences exist in lipid profiles and in the prevalence of dyslipidemia. However, these studies did not provide information about Asian children.

The 50th, 75th, 90th, and 95th percentile values for cholesterol concentrations from the KNHANES IV adolescents aged 10 to 18 years were 152, 171, 191, and 200 mg/dL, respectively, for boys and 160, 176, 194, and 205 mg/dL, respectively, for girls. In comparison, the percentile values calculated from the United States NHANES 1999 to 2006 data for white boys aged 6 to 17 years were 157, 177, 195, and 212 mg/dL, respectively, and those for white girls were 162, 182, 201, and 219 mg/dL, respectively [15]. Thus, Korean children and adolescents showed less hypercholesterolemia than white children did from the United States (6.5% vs. 9.3%). In addition, the 50th, 75th, 90th, and 95th percentiles for LDL-C concentrations were 87, 104, 119, and 127 mg/dL, respectively, for Korean boys and 90, 106, 121, and 132 mg/dL, respectively, for girls. The corresponding numbers for white boys from the United States were 85, 103, 124, and 136 mg/dL, respectively, and those for girls were 88, 104, 122, and 133 mg/dL, respectively [15]. The mean and 50th percentiles were similar between Koreans and white individuals from the United States, but the 95th percentile values for Koreans were less than those obtained for white individuals from the United States. Thus, Korean children and adolescents showed less incidence of high LDL-C dyslipidemia than white from the United States (4.7% vs. 7.7%).

The TG profile also showed a similar pattern. The 50th and 95th percentiles for TG concentrations were 73 and 189 mg/dL for Korean boys and 80 and 181 mg/dL for girls. In comparison, the percentiles calculated from the United States NHANES data from 1988 to 1994 for white boys 12 to 19 years of age were 76 and 205 mg/dL, respectively, and those for white girls were 80 and 218 mg/dL, respectively. The 5th and 50th percentiles for HDL-C concentrations were 35 and 48 mg/dL for Korean boys and 37 and 50 mg/dL for girls. In comparison, the percentiles calculated from the NHANES 1988 to 1994 data for white boys aged 4 to 19 years were 37 and 53 mg/dL, respectively, and those for white girls were 36 and 54 mg/dL, respectively [14]. Korean children and adolescents showed less incidence of high TG but higher incidence of low HDL-C than that shown by white in the United States (10.1% vs. 12.1%; 7.1% vs. 8.5%). However, the prevalence of total dyslipidemia (at least one abnormal lipid concentration) in Korean children and adolescents according to guidelines of the NCEP and AHA was 19.7%, which was comparable with the value (20.3%) for subjects from the United States [20]. That might be due to single high TG or low HDL-C in Korean subjects, as previously mentioned [12], [21]. Nonetheless, the 95th percentiles for TC and LDL-C and the 5th percentile for HDL-C in Korean were 203 mg/dL, 129 mg/dL, and 49 mg/dL, respectively. The 90th percentile for TG concentrations was 150 mg/dL. Thus, it would be suitable for Korean children and adolescents to follow the AAP guidelines for dyslipidemia to prevent CVD in adulthood by lifestyle modification or medical intervention.

Gender differences in lipid concentrations also existed in Korean children and adolescents, as reported in other previous studies [14], [15]. Girls had higher TC and LDL-C than did boys. Girls also tended to have higher HDL-C than did boys did after the age of 12–13 years, which corresponded to the period of pubertal development. Thus, girls had a higher prevalence of hypercholesterolemia and high LDL-C, which was different from the findings in their white counterparts in the United States. In the United States, boys showed a higher prevalence of hypercholesterolemia and high LDL-C. This phenomenon is observed in other Asian countries as well [22], [23]. The prevalence of hypercholesterolemia and high LDL-C in females is higher than those in males in subjects older than 50 years of age in Korea and China [12], [24]. Asian females may have a higher prevalence of dyslipidemia in both adolescence and after menopause.

In Korea, like in other Asian countries, the prevalence of CVD was much lower than that in Western countries 50 years ago [25]. However, cardiovascular disease morbidity and mortality in Korea has been projected to increase both in absolute number and as a proportion of total disease burden, and the mortality rate associated with CVD increased up to 27.6% in a recent assessment [2], [12]. This trend is common in all East-Asian countries [26]. However, CVD and its mortality rate in US have declined by more than 56 percent [27]. That might partially be due to the availability of drug and non-drug strategies that could reduce CVD. The prevalence of hypercholesterolemia, high LDL-C, and high TG continue to decrease in the United States in spite of relative increase in obesity [15], [28]. However, plasma lipid concentrations increased in Korean children like in other Asian populations [29]–[31]. In addition, the detection rate of hypercholesterolemia increased from 9.1% to 21.3% in Japanese children similar to Korean [29]. One of the reasons could be the westernized lifestyle changes with the economic development in these regions, especially the consumption of diets rich in fat, sugar, and cholesterol [29]–[31].

Dyslipidemia is one of the most important modifiable risk factors for cardiovascular diseases [5], [11]. Although ethnic differences exist in the prevalence of dyslipidemia, the estimated 10-Year Risk of Coronary Heart Disease does not differ according to ethnicity [32]. Asians are known to have the same risk of CVD in spite of the lower prevalence of dyslipidemia [12], [26]. Furthermore, Asians have other risk factors for CVD, such as hypertension and diabetes. In our study, the prevalence of hypertension was up to 19.2% and in hypertensive subjects, the relative risk of dyslipidemia increased by 1.474 times. Furthermore, the age-adjusted prevalence of hypertension in Korean adults was 34.7%, which is higher than that (29.5%) in the United States [33], [34]. The prevalence of type 2 diabetes mellitus in Korea is estimated to be 7.3%, according to a report by KNHANES III, 2005, an approximately five-fold increase from the value of 30 years ago [35],[36]. The estimated prevalence of diabetes mellitus among Koreans is even higher than in the U.S. population in 2030. Furthermore, 0.09% of children and adolescents were newly diagnosed with diabetes, which was comparable to the findings in Asians in the SEARCH for Diabetes in Youth Study. [37] The risk of CVD estimated from the NCEP guideline and predicted using the Framingham model in Koreans was similar to that in the American population (Korea, 4.7% vs. the U.S., 5.3%) [38]. Therefore, adult dyslipidemia patients have been treated according to NCEP or AHA guidelines with some modifications [39]. On the basis of these and our own results, we suggest that dyslipidemia in Korean and other Asian children and adolescents should be managed according to the recommendations in the AAP guidelines.

There were some limitations in the present study. First, we were unable to estimate precisely the number of children and adolescents who were potentially eligible for pharmacological treatment because information regarding family history of CVD was not collected in KNHANES-IV. Second, the subject who was suspected diabetes with high LDL-C and high HbA1c was not included as they were not fasting state. If they were included the adolescent potentially eligible for pharmacological treatment would further increase.

### Conclusion

We made the references of each lipid and lipoprotein concentrations in Korean children and adolescents aged 10–18 years. The estimated national prevalence of any type of pediatric dyslipidemia by using the NECP and AHA guidelines was 19.7%. Furthermore, at least 0.41% of Korean children and adolescents are eligible for pharmacological treatment according to the AAP guidelines. These findings provide useful information not only for Korean but also for Asian ethnicity in planning programs targeting the prevention of CVD through lipid control from childhood.
